# NOTCH2 participates in Jagged1-induced osteogenic differentiation in human periodontal ligament cells

**DOI:** 10.1038/s41598-020-70277-7

**Published:** 2020-08-07

**Authors:** Jeeranan Manokawinchoke, Piyamas Sumrejkanchanakij, Lawan Boonprakong, Prasit Pavasant, Hiroshi Egusa, Thanaphum Osathanon

**Affiliations:** 1 Center of Excellence for Regenerative Dentistry and Department of Anatomy, Faculty of Dentistry, Chulalongkorn University, Bangkok, 10330 Thailand; 2 Oral Biology Research Center, Faculty of Dentistry, Chulalongkorn University, Bangkok, 10330 Thailand; 3 Division of Molecular and Regenerative Prosthodontics, Tohoku University Graduate School of Dentistry, Sendai, 980-8575 Japan; 4 Genomics and Precision Dentistry Research Unit, Faculty of Dentistry, Chulalongkorn University, Bangkok, 10330 Thailand

**Keywords:** Stem-cell differentiation, Osteoblasts

## Abstract

Jagged1 activates Notch signaling and subsequently promotes osteogenic differentiation in human periodontal ligament cells (hPDLs). The present study investigated the participation of the Notch receptor, NOTCH2, in the Jagged1-induced osteogenic differentiation in hPDLs. *NOTCH2* and *NOTCH4* mRNA expression levels increased during hPDL osteogenic differentiation. However, the endogenous NOTCH2 expression levels were markedly higher compared with NOTCH4. *NOTCH2* expression knockdown using shRNA in hPDLs did not dramatically alter their proliferation or osteogenic differentiation compared with the shRNA control. After seeding on Jagged1-immobilized surfaces and maintaining the hPDLs in osteogenic medium, *HES1* and *HEY1* mRNA levels were markedly reduced in the sh*NOTCH2*-transduced cells compared with the shControl group. Further, sh*NOTCH2*-transduced cells exhibited less alkaline phosphatase enzymatic activity and in vitro mineralization than the shControl cells when exposed to Jagged1. *MSX2* and *COL1A1* mRNA expression after Jagged1 activation were reduced in sh*NOTCH2-*transduced cells. Endogenous Notch signaling inhibition using a γ-secretase inhibitor (DAPT) attenuated mineralization in hPDLs. DAPT treatment significantly promoted *TWIST1*, but decreased *ALP*, mRNA expression, compared with the control. In conclusion, Notch signaling is involved in hPDL osteogenic differentiation. Moreover, NOTCH2 participates in the mechanism by which Jagged1 induced osteogenic differentiation in hPDLs.

## Introduction

Notch signaling regulates various cell responses during development and homeostasis, including cell proliferation, survival, and differentiation. The binding of a transmembrane Notch ligand to a Notch receptor on adjacent cells activates intracellular Notch signaling by releasing the Notch intracellular domain (NICD) of the receptor. The NICD translocates into the nucleus and binds to a transcriptional complex, turning this transcriptional repressor to an activator complex. The mammalian canonical Notch receptors and ligands are NOTCH1, NOTCH2, NOTCH3, NOTCH4, and DLL1, DLL3, DLL4, JAG1, and JAG2, respectively^1,2^.

The role of Notch signaling in dental-related cells has been reported. Notch signaling directly and indirectly regulates osteoclastogenesis. In orthodontic-treated rodent teeth, Notch receptors and ligands are upregulated in bone resorptive sites^3^. Jagged1 decreases osteoprotegerin (*OPG*), but not receptor activator of nuclear factor-κB ligand (*RANKL*), expression in human periodontal ligament cells (hPDLs)^4^. Hence, the OPG/RANKL ratio is reduced, favoring osteoclast formation. Correspondingly, Jagged1 directly influences osteoclast differentiation in a murine macrophage-like cell line^5^. Moreover, Notch signaling increases PDL cell viability under stress^6^. Jagged1 immobilization activates Notch signaling and promotes osteogenic differentiation in hPDLs^7,8^. Jagged1-treated hPDLs exhibit increased alkaline phosphatase (ALP) enzymatic activity and an in vitro mineral deposition compared with cells in the control condition. Furthermore, osteogenic medium upregulates the mRNA expression of the Notch ligand, *DLL1*, in hPDLs^9^. Adding a Notch signaling inhibitor attenuates osteoblast maturation as determined by decreased mineral deposition^10^. Taken together, these data suggest the role of Notch signaling in hPDL homeostasis.

A previous study demonstrated that hPDLs express all 4 Notch receptors, *NOTCH1-4*^4^. The extracellular domain of the Notch receptor consists of epidermal growth factor (EGF)-like repeats that function as ligand binding sites. Different Notch receptors contain different numbers of repeats and exhibit different ligand binding properties and preferentially bind with certain ligands^11^. It has been reported that each Notch receptor influences different cell responses^11^. Notch receptors are expressed in specific locations during organ development and mutation of each receptor leads to dissimilar phenotypes^11–13^. Hence, the specific roles of each Notch receptor should be determined. The present study investigated the participation of NOTCH2 in Jagged-induced osteogenic differentiation in hPDLs.

## Results

### Notch receptors and ligands are expressed during hPDL osteogenic differentiation

**Figure 1:**
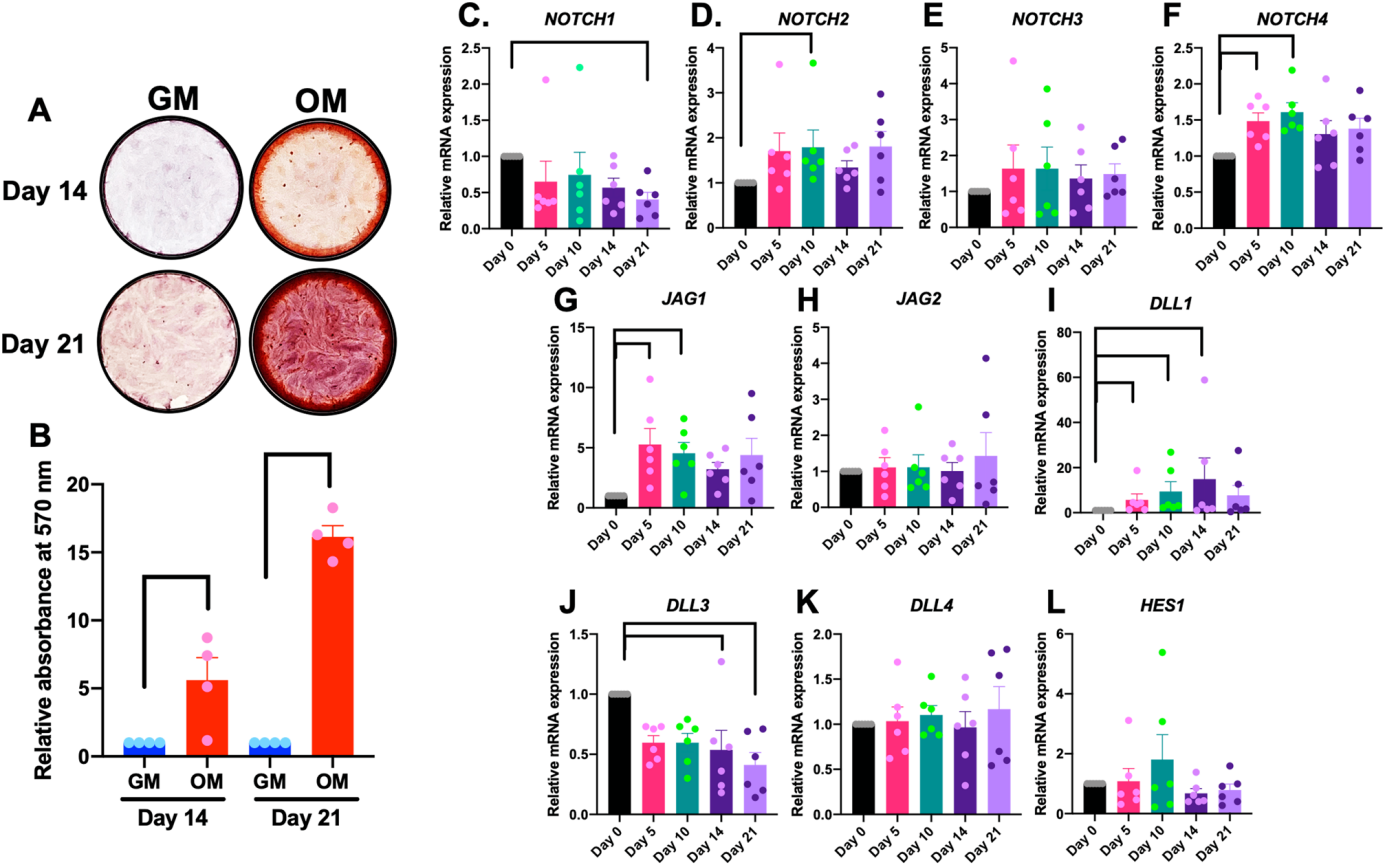
The mRNA expression of Notch receptors, ligands, and target genes during hPDL osteogenic differentiation. hPDLs were maintained in growth medium (GM) or osteogenic medium (OM). Mineral deposition was determined by alizarin red s staining at day 14 and 21 (**A**). The stained dye was solubilized and measured at an absorbance at 570 nm (**B**). The mRNA expression of Notch receptors, ligands, and target genes was evaluated by real-time PCR at day 0, day 5, day 10, day 14, and day 21 (**C**–**L**). Bars indicate a significant difference.

hPDLs differentiated into the osteogenic lineage when the cells were incubated in osteogenic medium (OM). A significant increase in mineral deposition was observed at 14 and 21 days in OM (Fig. 1A,B). The mRNA expression levels of Notch receptors, ligands, and target genes were examined using real-time PCR at day 0, day 5, day 10, day 14, and day 21 during osteogenic differentiation. Among the Notch receptors, *NOTCH3* mRNA levels did not change during the observation period. The increased expression of *NOTCH2* and *NOTCH4* was observed during osteogenic differentiation while *NOTCH1* mRNA levels significantly decreased at day 21 compared with day 0 (Fig. 1C–F). A significant increase from day 0 was noted at day 10 for *NOTCH2*, and at day 5 and day 10 for *NOTCH4*. A significant upregulation of Notch ligands (*JAG1* and *DLL1*) was detected at day 5 and day 10 for *JAG1* and at day 5, day 10, and day 14 for *DLL1* during osteogenic differentiation compared with day 0 (Fig. 1G–L). *DLL3* mRNA levels significantly decreased at day 14 and day 21 compared with day 0. In contrast, there was no marked difference in *JAG2, DLL4,* and *HES1* mRNA expression during osteogenic differentiation (Fig. 1G–L).

**Figure 2:**
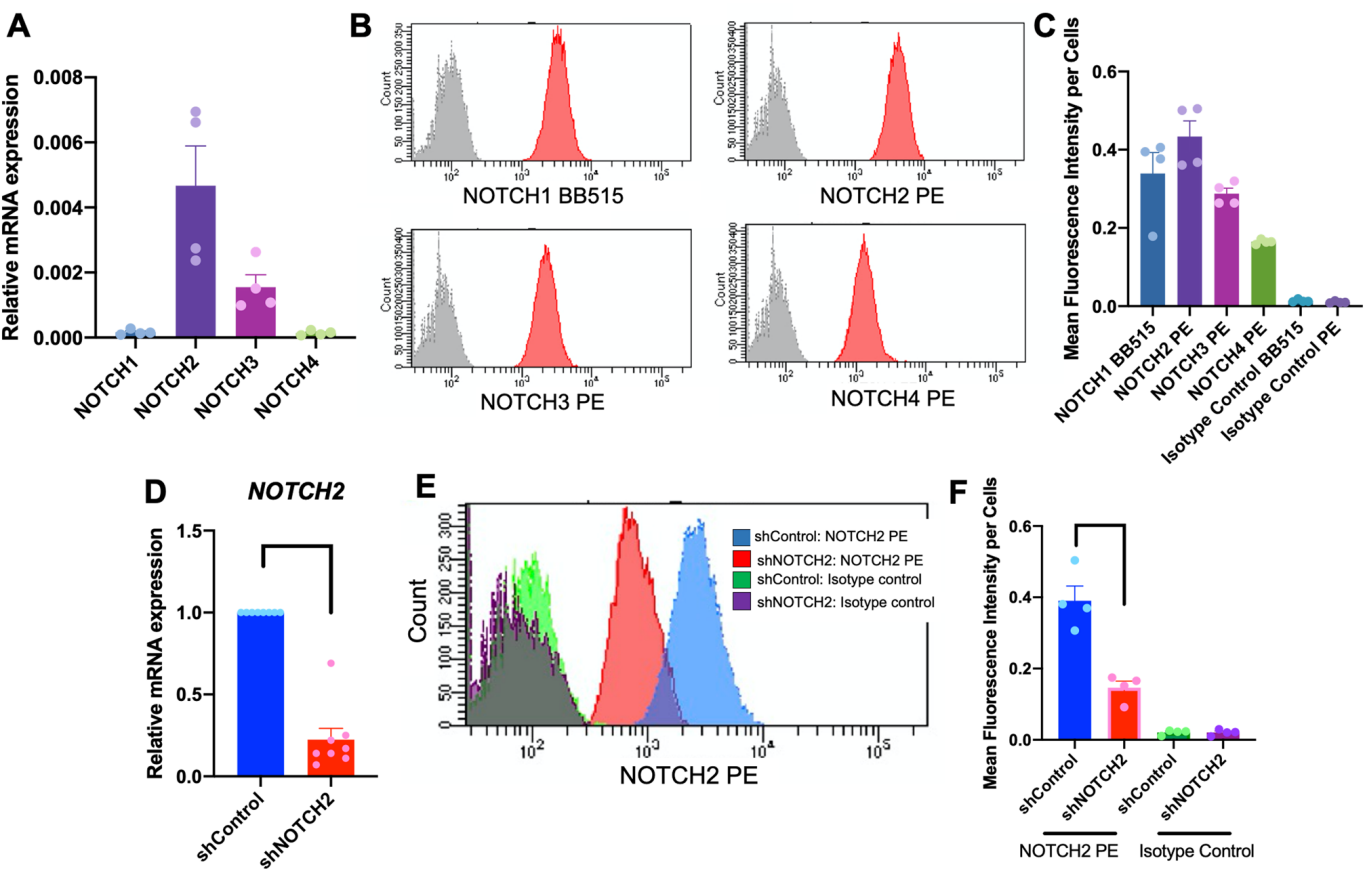
The baseline mRNA expression of the four Notch receptors in hPDLs was examined using real-time PCR (**A**). Protein expression levels were examined using flow cytometry analysis. The representative histogram was illustrated (**B**) and the mean fluorescence intensity per cells was demonstrated (**C**). Cells were transduced with either lentiviral particles containing shRNA scramble sequence (shControl) or shRNA against *NOTCH2* (shNOTCH2). The NOTCH2 knockdown efficiency was examined using real-time PCR (**D**) and flow cytometry analysis (E and F). Bars indicate a significant difference.

The baseline mRNA expression of Notch receptors was evaluated using real-time PCR. The results demonstrated that hPDLs expressed all four types of Notch receptors, with *NOTCH2* mRNA levels being the most highly expressed (Fig. 2A). Correspondingly, protein expression of all four types of Notch receptors was evaluated using flow cytometry analysis (Fig. 2B,C). NOTCH2 protein was the most expressed Notch receptor in hPDLs.

### NOTCH2 knockdown did not alter cell proliferation or mineralization

**Figure 3:**
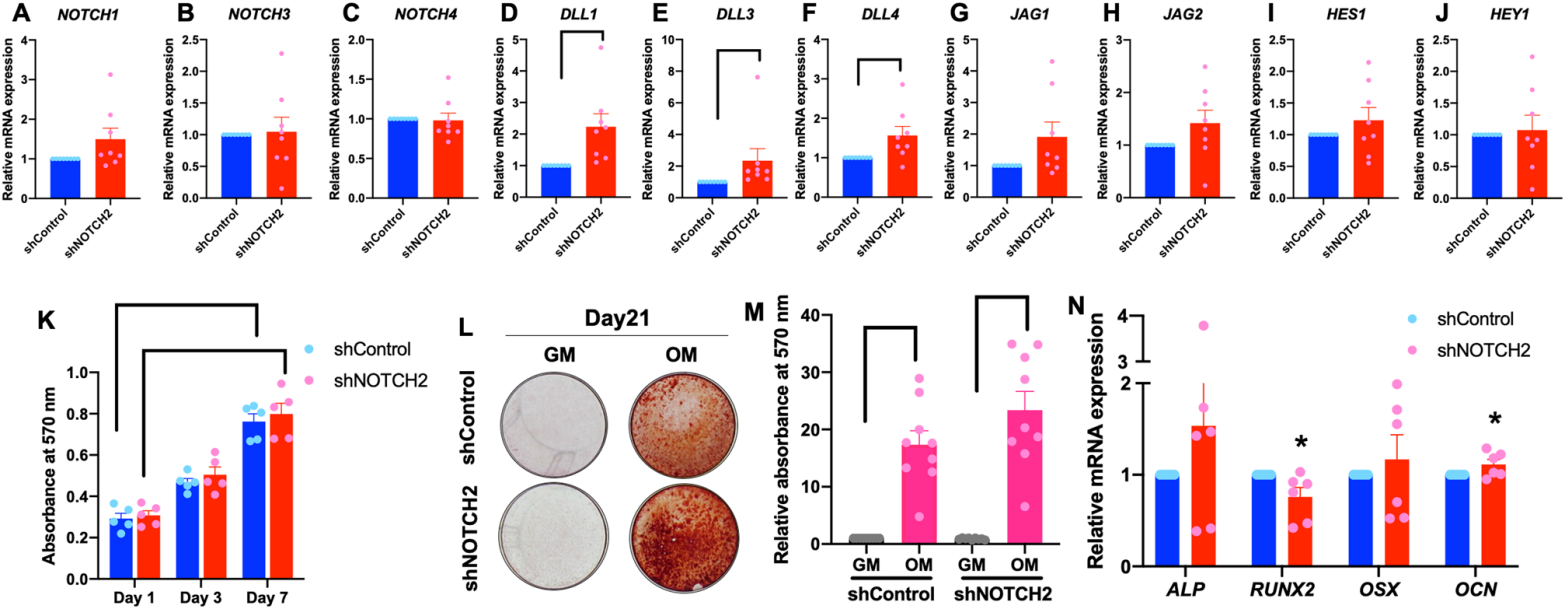
Effect of *NOTCH2* knockdown on cell proliferation and osteogenic differentiation in hPDLs. Cells were transduced with either lentiviral particles containing shRNA scramble sequence (shControl) or shRNA against *NOTCH2* (shNOTCH2). The mRNA expression of Notch pathway components was examined using real-time PCR (**A**–**J**). Cell viability was determined at day 1, 3, and 7 days (**K**). shControl and shNOTCH2 cells were maintained in either growth medium or osteogenic medium for 21 days and mineral deposition was stained with alizarin red s dye (**L**). Absorbance values of solubilized dye (**M**). Osteogenic marker gene expression was evaluated using real-time PCR at day 7 after osteogenic differentiation (**N**). Bars indicate a significant difference. Asterisks indicate a significant difference compared with shControl.

Because NOTCH2 was the highest expressed Notch receptor in hPDLs and significantly increased during osteogenic differentiation, hPDLs were subjected to knockdown of *NOTCH2* expression using shRNA. The hPDLs were transduced with lentiviral particles containing shRNA against *NOTCH2* (shNOTCH2). Lentiviral particles containing a scrambled shRNA sequence were employed in the control condition (shControl). PCR and flow cytometry analysis were performed to confirm the NOTCH2 knockdown. A significant reduction in *NOTCH2* mRNA and protein levels was observed in shNOTCH2 cells (Fig. 2D–F). NOTCH2 mRNA and protein expression were 22.38% and 37.4% compared with the control condition, respectively. There was no significant difference in other Notch receptor mRNA levels between the shControl and shNOTCH2 groups (Fig. 3A–C). A significant increase in *DLL1, DLL3,* and *DLL4* mRNA levels was detected (Fig. 3D–F). However, *JAG1*, *JAG2, HES1,* and *HEY1* mRNA expression were similar between the groups (Fig. 3G–J).

Both shControl and shNOTCH2 cells demonstrated a significant increase in cell number at day 7 compared with day 1 (Fig. 3K). However, there was no significant difference between these two groups at the observed time points. When the cells were maintained in OM for 21 days, both shControl and shNOTCH2 cells formed mineral deposits in OM (Fig. 3L). A significant increase in mineral precipitation was present in OM compared with growth medium (GM); however, there was no dramatic change in mineralization in the shNOTCH2 group compared with the shControl group (Fig. 3M). Correspondingly, the *ALP* and *OSX* mRNA levels in the cells maintained in OM for 7 days were similar between the shControl and shNOTCH2 groups (Fig. 3N). Although there was a significant difference in *RUNX2* and *OCN* mRNA levels in the shNOTCH2 group compared with the shControl group, the change was less than 0.25-fold.

### NOTCH2 participated in Jagged1-induced osteogenic differentiation in hPDLs

**Figure 4:**
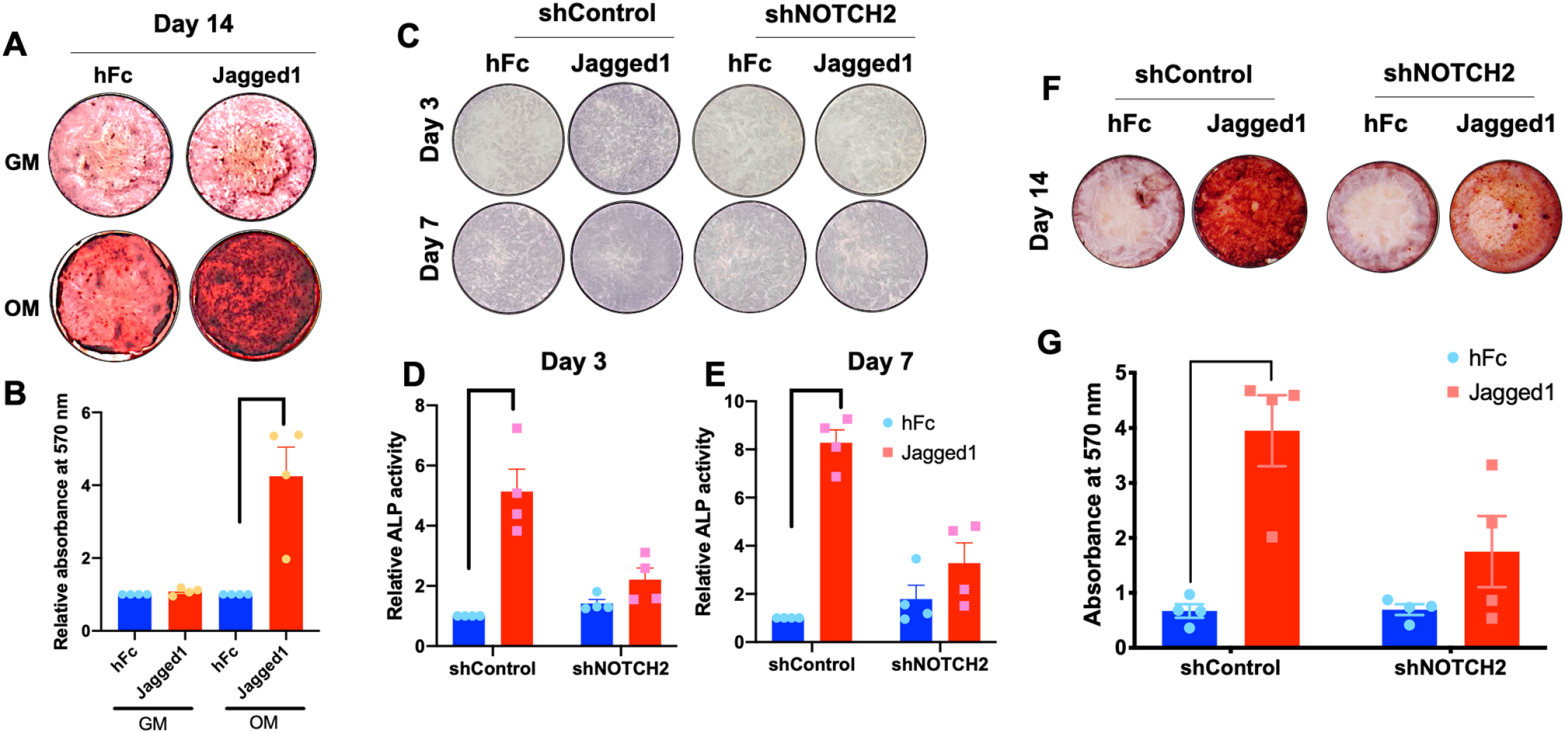
shNOTCH2 cells exhibited less ALP enzymatic activity and mineralization in Jagged1 treated condition. hPDLs were seeded on Jagged1-immobilized surfaces. The control condition was hFc-immobilized surfaces. Mineral deposition was examined using alizarin red s staining at day 14 (**A**). Absorbance of the solubilized dye (**B**). ALP staining and enzymatic activity assay were evaluated at day 3 and 7 after osteogenic differentiation (**C**–**E**). An in vitro mineralization was stained with alizarin red s dye (**F**). Absorbance of the solubilized dye (**G**). Bars indicate a significant difference. *GM* growth medium, *OM* osteogenic medium.

Previous reports demonstrated that indirect affinity-immobilized Jagged1 promoted osteogenic differentiation in hPDLs as determined by increased ALP activity, osteogenic marker gene expression, and mineralization^7,8,14^. The hPDLs seeded on Jagged1 surfaces demonstrated dramatically enhanced an in vitro mineralization compared with those on the hFc control surfaces when the cells were maintained in OM for 14 days (Fig. 4A,B).

**Figure 5:**
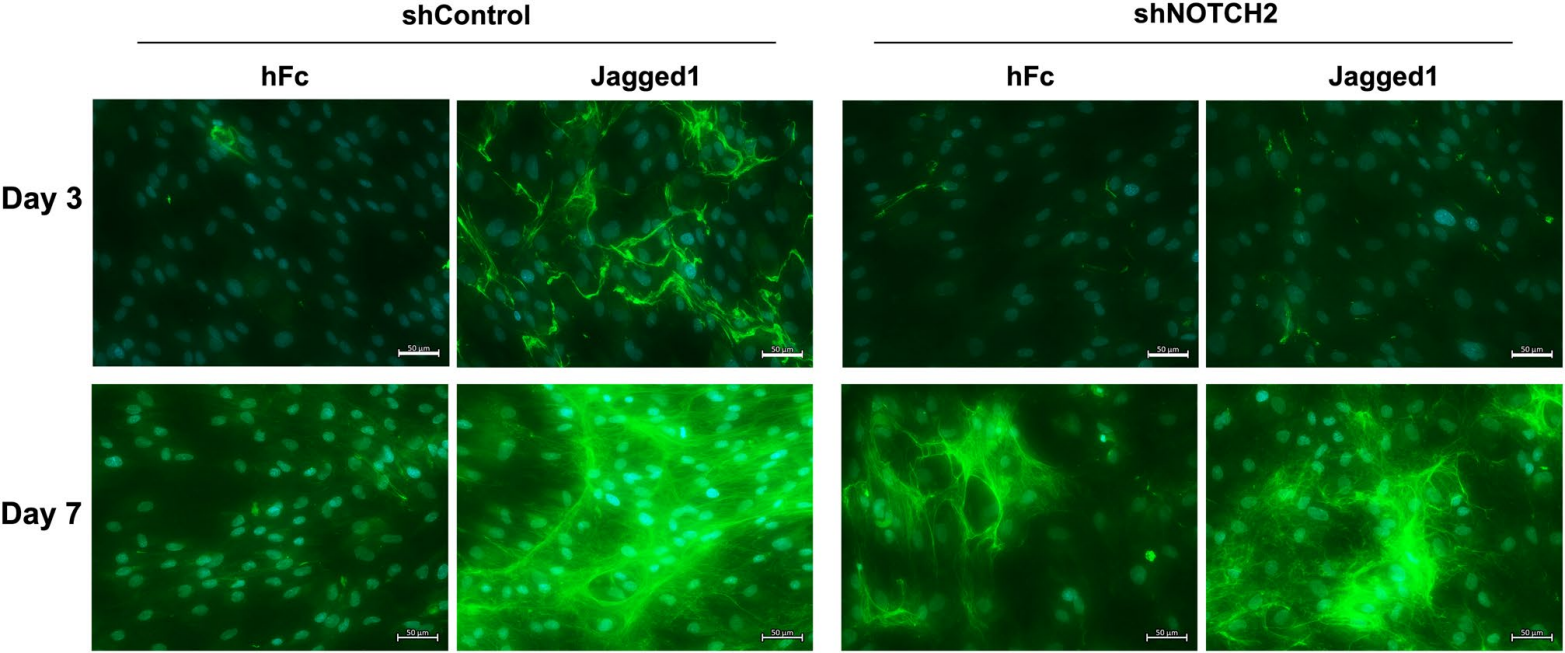
shNOTCH2 cells expressed less collagen type I in Jagged1 treated condition. hPDLs were seeded on Jagged1-immobilized surfaces. The control condition was hFc-immobilized surfaces. Collagen type I protein expression was examined using immunofluorescence staining at day 3 and day 7 after osteogenic differentiation. White bars indicate 50 μm.

shControl and shNOTCH2 cells were seeded on Jagged1-immobilized surfaces and cultured in OM. ALP enzymatic activity and osteogenic marker gene expression were evaluated at day 3 and day 7. Mineral deposition was examined using alizarin red s staining, SEM, and EDX at day 14. Jagged1-induced ALP enzymatic activity was markedly increased in the shControl, but not in the shNOTCH2 cells at both day 3 and day 7 (Fig. 4C–E). In addition, Jagged1-induced mineral deposition was attenuated in the shNOTCH2 cells, while the shControl cells on Jagged1 surfaces exhibited a substantial increase in mineralization compared with those on hFc control surfaces (Fig. 4F,G). Jagged1-induced COL1 protein expression was markedly attenuated in shNOTCH2 cells compared with those detected in the shControl cells at both day 3 and day 7 after osteogenic differentiation (Fig. 5).

**Figure 6:**
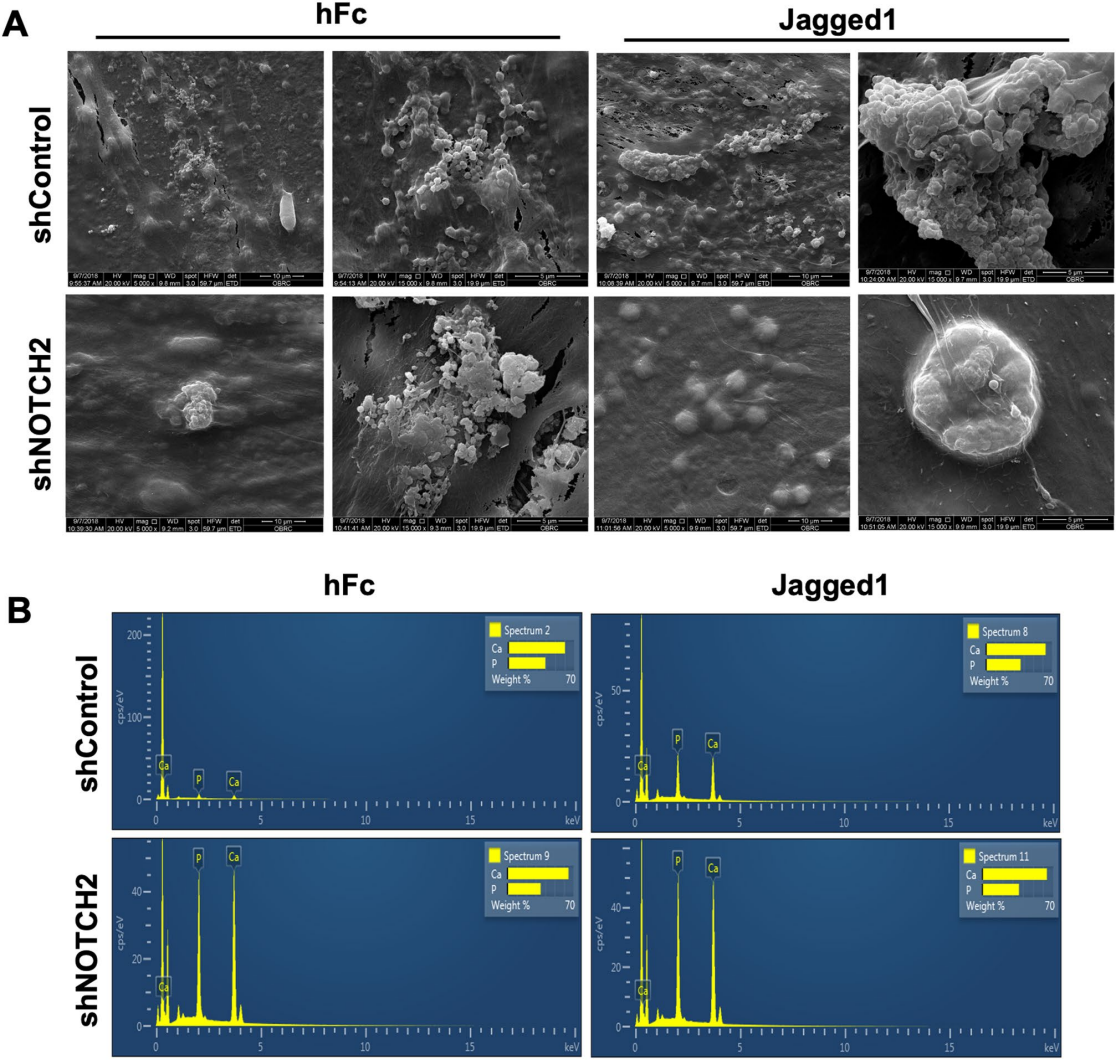
shNOTCH2 cells exhibited less mineralization in Jagged1-treated condition. shControl and shNOTCH2 cells were seeded on Jagged1-immobilized surfaces. The control condition was hFc-immobilized surfaces. Mineral crystal morphology and composition were determined using scanning electron microscope (**A**) and energy-dispersive X-ray spectroscopic analysis (**B**) at day 14 after osteogenic differentiation.

**Figure 7:**
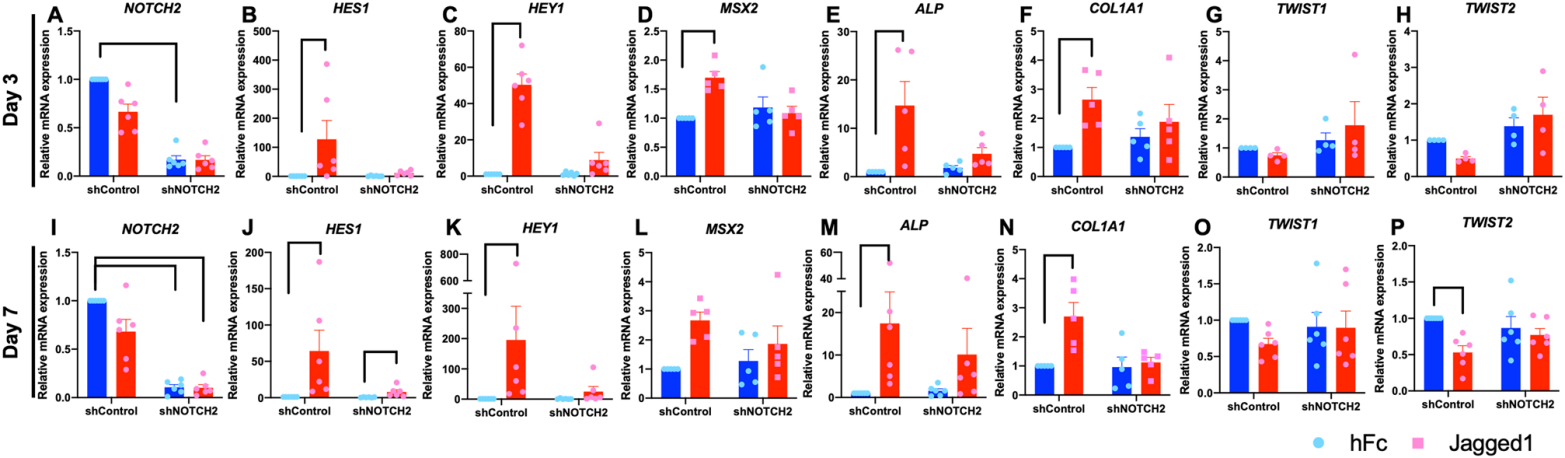
shNOTCH2 attenuated the effect of Jagged1 on Notch target gene and osteogenic marker gene expression. shControl and shNOTCH2 cells were seeded on Jagged1-immobilized surfaces. The control condition was hFc-immobilized surfaces. The mRNA expression was evaluated using real-time PCR at day 3 and day 7 after osteogenic differentiation. Bars indicate a significant difference.

The mineral crystals in Jagged1-treated shNOTCH2 cells were fewer and smaller compared with the Jagged1-treated shControl cells (Fig. 6A). However, the chemical composition of the mineral crystals was similar between the shControl and shNOTCH2 cells (Fig. 6B). The mRNA expression was examined at day 3 and day 7 (Fig. 7). Reduced *NOTCH2* mRNA levels in the shNOTCH2 cells were consistently evident compared with the shControl cells in OM (Fig. 7A,I). shNOTCH2 cells exhibited lower Notch target gene, *HES1* and *HEY1*, expression levels after exposure to Jagged1-immobilized surfaces (Fig. 7B,C,J,K). *HES1* and *HEY1* mRNA levels were markedly upregulated in Jagged1 treated shControl cells, however there was no significant difference in *HES1* and *HEY1* mRNA expression in Jagged1-treated shNOTCH2 cells compared with the hFc control. *MSX2, ALP,* and *COL1A1* mRNA levels were significantly enhanced in the shControl cells on Jagged1 surfaces compared with those on hFc surfaces (Fig. 7D,F,L,N). A negative regulator of osteogenic differentiation, *TWIST2*, mRNA levels were significantly decreased in Jagged1-treated shControl cells (Fig. 7G,H,O,P). None of the osteogenic maker genes were significantly altered in shNOTCH2 cells in the Jagged1 condition compared with those in the hFc condition.

### Notch signaling inhibition attenuated mineralization in hPDLs

**Figure 8:**
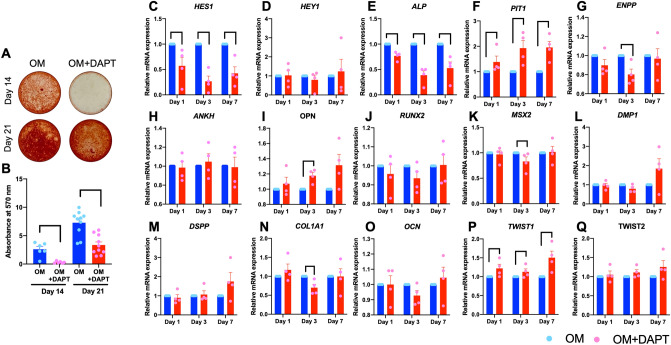
Endogenous Notch signaling participated in mineralization in hPDLs. hPDLs were maintained in osteogenic medium (OM) with or without a γ-secretase inhibitor (DAPT). Mineralization was determined using alizarin red s staining at day 14 and 21 (**A**). The absorbance at 570 nm of the solubilized dye (**B**). The mRNA expression was examined using real-time PCR at day 1, 3, and 7 (**C**–**Q**). Bars indicate a significant difference.

To evaluate the role of endogenous Notch signaling in hPDL osteogenic differentiation, cells were maintained in OM with or without a γ-secretase inhibitor, DAPT. DAPT treatment attenuated mineral deposition in hPDLs at 14 and 21 days after osteogenic differentiation (Fig. 8A,B). *HES1,* but not *HEY1,* mRNA was significantly decreased in the DAPT-treated condition at the observed time points (Fig. 8C,D). Because DAPT decreased mineralization in hPDLs, the mRNA expression of the phosphate regulatory genes was investigated. DAPT-treatment significantly decreased *ALP*, but increased *PIT1* mRNA levels, at all time points (Fig. 8E,F). *ENPP* mRNA expression was significantly decreased at day 3, while there was no change in *ANKH* mRNA levels in the DAPT-treated group (Fig. 8G,H). *OPN* mRNA expression increased at all time points, however, the difference was signficant only at day 3 (Fig. 8I). For osteogenic-related transcription factors, hPDLs treated with DAPT demonstrated similar *RUNX2* mRNA levels compared with the control condition, while markedly reduced *MSX2* expression levels were observed at day 3 (Fig. 8J,K). There was no significant change in the mRNA levels of *DMP1, DSPP,* or *OCN* in the DAPT-treated group compared with the control (Fig. 8L,M,O) . However, decreased *COL1A1* expression levels were noted at day 3 (Fig. 8N). *TWIST1,* but not *TWIST2,* mRNA levels were significantly upregulated in the DAPT-treated hPDLs (Fig. 8P,Q).

**Figure 9:**
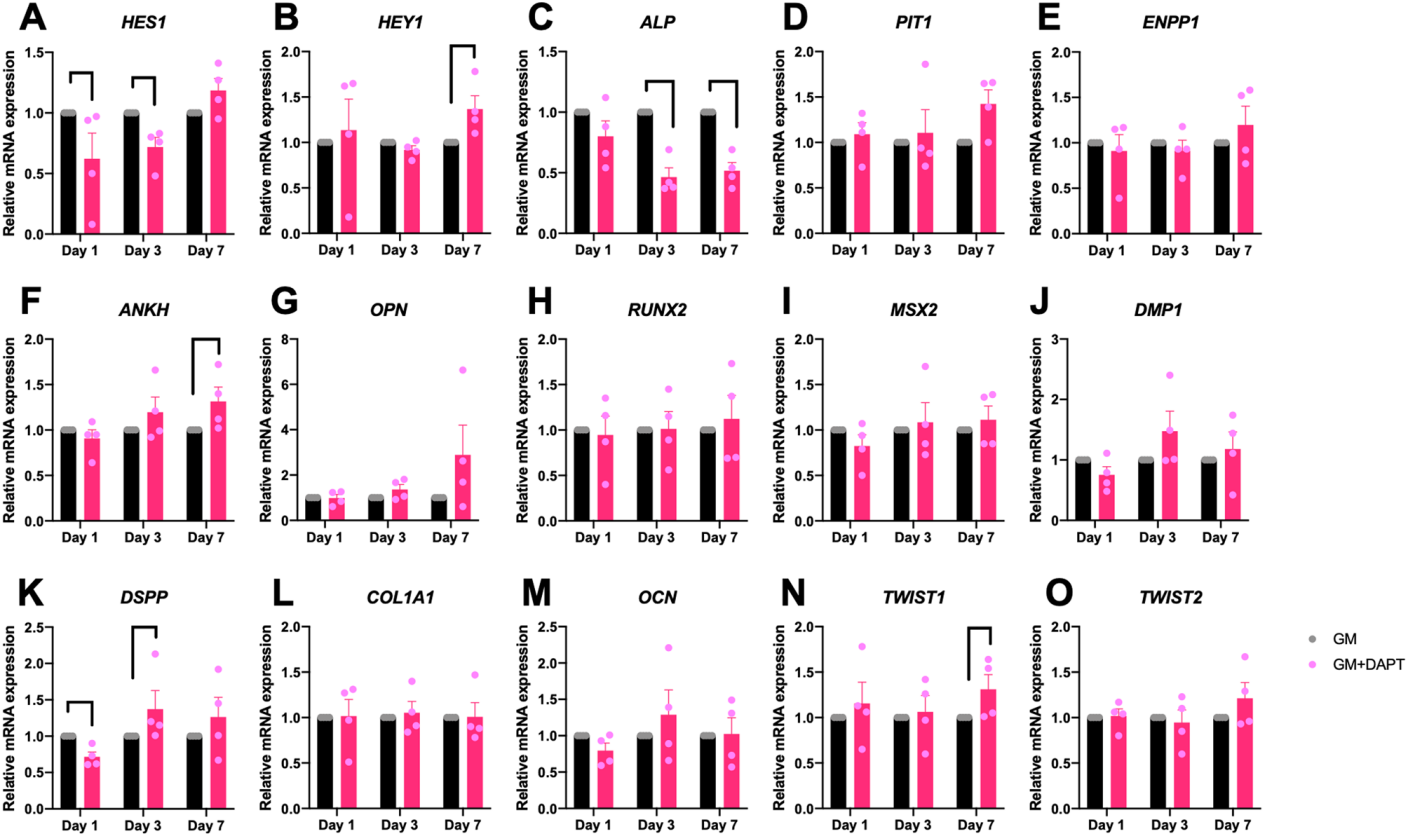
Notch signaling inhibition in growth medium attenuated *ALP* mRNA expression. hPDLs were maintained in the control growth medium (GM) with or without a γ-secretase inhibitor (DAPT). The mRNA expression was examined using real-time PCR at day 1, 3, and 7 (**A**–**O**). Bars indicate a significant difference.

Effect of endogenous Notch signaling inhibition on osteogenic related gene expression was also evaluated in the GM. hPDLs were cultured in GM and treated with DAPT for 1, 3, and 7 days. Results demonstrated that DAPT was able to inhibit *HES1* mRNA expression, confirming Notch signaling inhibition (Fig. 9A). DAPT treated hPDLs in growth medium significantly decreased *ALP* mRNA levels (Fig. 9C). Although, there were significant difference of *ANKH, DSPP,* and *TWIST1* mRNA levels between the control and DAPT treated condition. The fold change was less than 1.5 (Fig. 9).

## Discussion

The present study described the role of NOTCH2 in immobilized Jagged1-induced osteogenic differentiation in hPDLs. Previous report demonstrated that Jagged1 promoted osteogenic differentiation in hPDLs. In the present study, we described in detail on the receptor participating in this regulation. *NOTCH2* mRNA and protein levels is the highest among Notch receptors in hPDLs and significantly increased during osteogenic differentiation. *NOTCH2* knockdown resulted in the marked decrease of ALP enzymatic activity, collagen type I expression, and mineral deposition. Hence, the present study highlights the participation of NOTCH2 in mechanism by which Jagged1 induced osteogenic differentiation in hPDLs.

Notch signaling regulates osteogenic differentiation. However, this effect is cell type and stage specific^15^. The Notch ligand, Jagged1, markedly promoted osteogenic differentiation in hPDLs as determined by a significant increase in ALP enzymatic activity, osteoblast marker gene expression, and mineral deposition in vitro^8,16^*.* Corresponding, Jagged1-induced osteogenic differentiation was observed in human mesenchymal stem cells (hMSCs), human dental pulp cells, stem cells isolated from human exfoliated deciduous teeth, and human bone-derived cells^7,15,17–20^. In contrast, Jagged1 did not dramatically promote osteogenic differentiation in retinoic acid treated mouse induced pluripotent stem cells^21^. The mechanism(s) related to this phenomenon remains unclear. It was shown that a PKCδ inhibitor attenuated Jagged1-induced osteogenic differentiation in hMSCs^15^, implying that PKCδ participates in the regulatory mechanism. A study in vascular smooth muscle cells illustrated that the Notch1 intracellular domain enhanced osteogenic differentiation via increased *Msx2* transcriptional activity^22^. The present study demonstrated the involvement of NOTCH2 in Jagged1-induced *MSX2* expression in hPDLs. The shControl cells upregulated *MSX2* mRNA expression after exposure to Jagged1, while this phenomenon was attenuated in Jagged1-treated shNOTCH2 cells.

A previous study in human periodontal ligament stem cells (hPDLSCs) showed that Jagged1 promoted osteogenic differentiation by downregulating a negative regulator of osteogenic differentiation. Jagged1-treated hPDLSCs had a marked reduction of *TWIST* family gene expression^8^. However, the direct relation of Notch receptors and TWIST promoter activity has not been reported. A Twist/Notch correlation has been demonstrated in *Drosophila.* Notch activation caused Twist expression^23^. *Drosophilla* with reduced Twist or Notch expression levels exhibited similar muscle development phenotypes^23^. TWIST1 and TWIST2 also function as negative regulators of osteogenic differentiation by directly binding to the *RUNX2* promoter, inhibiting *RUNX2* transcription^24^. *TWIST* knockdown in hPDLs resulted in the increased mRNA expression of osteogenic marker genes and alkaline phosphatase enzymatic activity^25^. The present study demonstrated that endogenous Notch signaling inhibition resulted in increased *TWIST1* mRNA levels in hPDLs. Correspondingly, *TWIST2* mRNA expression was attenuated in Jagged1-treated shControl cells, while this effect was rescued in *NOTCH2* knockdown cells, confirming that Notch promotes osteogenic differentiation by downregulating *TWIST* gene expression via Jagged1/Notch2 binding.

hPDLs expressed all 4 types of canonical Notch receptors, NOTCH1-4. *NOTCH2* mRNA and protein levels were the highest among the Notch receptors. This result is consistent with previous studies showing high expression of *NOTCH2* in hPDL cells^4,26^. Different types of Notch receptors function differently when binding to the same ligand^27,28^. NOTCH2 plays a role in hPDL homeostasis, because it has been shown that orthodontic force application in rat molars led to upregulated Notch2 expression in rat periodontal tissues^3^. *NOTCH2* knockdown in hPDLs decreased the effect of Jagged1-induced osteoprotegerin expression^4^. In addition, *NOTCH2* mRNA expression was significantly increased after hPDLs were exposed to an intermittent compressive force for 24 h^26^. The upregulation of *NOTCH2* under intermittent stress also participated in the mechanism by which TGF-β1 regulated sclerostin expression in hPDLs^26^. However, the role of NOTCH2 in hPDL osteogenic differentiation remains unknown.

The function of Notch2 in osteogenic differentiation and bone formation is unresolved. Conditional knockout of *Notch2* driven by *Runx2* expression in cells in mice resulted in increased trabecular bone mass, implying that *Notch2* inhibits trabecular bone formation^29^. In contrast, a time course study in an MG63 osteosarcoma cell line found that *NOTCH1* and *NOTCH3* expression decreased in the early phase and *NOTCH2* and *NOTCH4* expression increased in the later phase during osteogenic differentiation in vitro^30^. Correspondingly, the present study in hPDLs observed a slight decrease in *NOTCH1* expression during the osteogenic differentiation time course, while *NOTCH2* and *NOTCH4* mRNA levels were upregulated. *NOTCH1*, *NOTCH2*, and *NOTCH4* mRNA levels were upregulated in human alveolar bone-derived osteoprogenitor cells seeded on modified titanium surfaces that correlated to increased osteogenic marker gene expression, implying the involvement of these receptors in osteogenic differentiation^31^. Previous research using human dental pulp cells illustrated that *HES1* expression increased during odonto/osteogenic differentiation^19^. The present study also observed a slight increase in *HES1* mRNA levels during hPDL osteogenic differentiation; however, the increase was not significant. This evidence suggests that Notch signaling positive regulates hPDL osteogenic differentiation.

shNotch2 cells exhibited the impairment of Jagged1-induced osteogenic differentiation. However, *NOTCH2* knockdown did not markedly affect osteogenic differentiation in hPDLs in condition without immobilized Jagged1, despite the fact that hPDLs expressed *JAG1*. As mentioned earlier, Notch signaling regulating cell response is complex and context dependent. Several hypotheses are postulated to address this point. First, we suspect that endogenous Notch signaling is dispensable during osteogenic differentiation process in hPDLs. Despite the fact that endogenous Notch signaling inhibition attenuated mineralization, DAPT treatment did not dramatically alter the expression levels of key osteogenic related transcriptional factors, *RUNX2* and *MSX2*. Although, the significant increase of *TWIST1* mRNA levels was observed. The fold change was less than 1.5. Second, hPDLs expressed both NOTCH2 and Jagged1. We hypothesize that endogenous Jagged1 protein expression levels are low, unlike the abundant Jagged1 immobilization on tissue culture surface. As Notch signaling initiates by the binding of transmembrane Notch receptor and ligand. There is no intracellular signaling amplification. Hence, the endogenous expression levels of both Notch receptors and ligands are indeed crucially impact on signal transduction and subsequently cell responses. Further study is necessitated to determine the endogenous protein levels of Jagged1 in hPDLs. Third, shNOTCH2 cells exhibited a slight change in Notch expression phenotypes. As shown in Fig. 3, shNOTCH2 cells significantly increased the expression of DLL ligands which could bind to other receptors and compensate the effect on osteogenic differentiation in shNOTCH2.

Inhibiting endogenous Notch signaling with a γ-secretase inhibitor (DAPT) resulted in attenuated mineral deposition without a marked change in osteogenic marker gene expression. It is hypothesized that endogenous Notch signaling controls phosphate/pyrophosphate metabolism and subsequently influence mineralization. DAPT suppressed *ALP* expression, while the *ANKH* mRNA levels did not change. This could result in a decreased phosphate/pyrophosphate ratio, which inhibits mineralization. In addition, *OPN* mRNA levels were increased in DAPT-treated hPDLs. OPN is an extracellular matrix component and considered an osteogenic differentiation marker. OPN is a potent mineralization inhibitor^32^. OPN binds to mineral crystals and prevents their growth^32^. Taken together, the potential reduced Pi/PPi ratio and increased *OPN* expression participate in the DAPT-attenuated mineralization in hPDLs.

In summary, the present study illustrated that *NOTCH2* participates in Jagged1-induced osteogenic differentiation in hPDLs because shNOTCH2-transduced cells exhibited the attenuated Jagged1-induced ALP enzymatic activity, osteogenic related gene expression, and mineral deposition compared with shControl cells. Further, direct evidence of the NOTCH2 intracellular domain interaction with the osteogenic related gene promotors is required to confirm the direct influence of NOTCH2.

## Materials and methods

### Cell isolation and culture

All experimental protocols and the study design was approved by the Human Research Ethics Committee, Faculty of Dentistry, Chulalongkorn University (approval no. 089/2015). All experimental protocols were carried out in accordance with the Helsinki declaration and relevant guideline and regulations. Informed consent was obtained. Embedded and impacted third molars without pathological conditions scheduled for surgical removal were collected for cell isolation. Periodontal ligament tissues were gently scraped from the middle 1/3 of the root to prevent gingival and cellular cementum cell contamination. The tissues were minced into small pieces and placed on 35-mm tissue culture dishes for cell explantation. The cells were maintained in growth medium (GM) composed of Dulbecco’s modified Eagle medium (DMEM, cat. no. 11960, Gibco, USA) containing 10% fetal bovine serum (FBS, cat. no. 10270, Gibco, USA), 2 mM L-glutamine (GlutaMAX-1, cat. no. 35050, Gibco, USA), 100 unit/mL penicillin, 100 μg/mL streptomycin and 250 ηg/mL amphotericin B (Antibiotic–Antimycotic, cat. no. 15240, Gibco, USA). The cells were incubated at 37 °C in a humidified 5% carbon dioxide atmosphere. The culture medium was changed every 48 h. The cells were sub-cultured at a 1:3 ratio when confluent. The experiments employed cells from passages 3‒6.

### Lentiviral shRNA transduction

Lentiviral shRNA transduction was performed in hPDLs with human *NOTCH2* shRNA lentiviral particles (cat. no. sc-40135-v; Santa Cruz Biotechnology, Dallas, TX, USA) or the control viral particle (cat. no. sc-108080; Santa Cruz Biotechnology). The transduction processes were performed according to the manufacturer’s protocol. Briefly, hPDLs were incubated in lentivirus transduction solution (5 × 10^4^ infectious units of virus) with 10 μg/mL polybrene (cat. no. sc-134220, Santa Cruz Biotechnology) for 18 h. The transduced hPDLs were selected with 10 μg/mL puromycin (cat. no. 108071, Santa Cruz Biotechnology).

### Osteogenic differentiation

hPDLs were seeded at a density of 75,000 cells/well on 24-well-plates and maintained in growth medium for 24 h. Subsequently, the culture medium was changed to osteogenic medium (OM), which was growth medium supplemented with 50 μg/mL ascorbic acid (cat. no. A-4034, Sigma-Aldrich, St. Louis, MO, USA), 250 nM dexamethasone (cat. no. D8893, Sigma-Aldrich), and 5 mM β-glycerophosphate (cat. no. G9422, Sigma-Aldrich). In some experiments, the osteogenic medium was supplemented with a γ-secretase inhibitor, DAPT (20 μM, cat. no. D5942, Sigma-Aldrich).

### Jagged1 treatment

Jagged1 was immobilized on the tissue culture surface using a previously described indirect affinity immobilization method^8^. Briefly, the tissue culture surfaces were incubated with recombinant protein G (50 μg/mL, cat. no. 101201, Invitrogen, Rockford, IL, USA) for 16 h, followed by coating the surface with bovine serum albumin solution (10 mg/mL, cat. no. A9418, Sigma-Aldrich) for 2 h. The tissue culture surfaces were rinsed with sterile phosphate buffered saline between steps. Lastly, the surfaces were exposed to Jagged1/Fc (10 nM, cat. no. 1277-JG, R&D Systems, Minneapolis, MN, USA) for 2 h. Human IgG Fc fragments (hFc, cat. no. 009000008, Jackson Immuno Research Labs, USA) were used in the control conditions.

### Polymerase chain reaction (PCR)

Total cellular RNA was isolated using Trizol reagent (RiboEx solution, cat. no. 301-001, GeneAll, Seoul, South Korea). Total RNA (1 μg) was converted to cDNA using reverse transcriptase (ImProm-II Reverse Transcription System, cat. no. A3800, Promega, Madison, WI, USA). Real-time PCR was performed in a CFX connect Real-Time PCR (Bio-Rad, Singapore) with FastStart Essential DNA Green Master (Roche Diagnostic, Mannheim, Germany). The real-time PCR reaction started with denaturing at 95 °C for 5 min, followed by 40 cycles of amplification, and ended with an extension reaction at 72 °C for 20 min. The amplification cycles were (1) 95 °C for 20 s, (2) 60 °C for 20 s, and (3) 72 °C for 20 s. Melt curve analysis was performed to determine product specificity. Relative gene expression was calculated using the 2^−ΔΔCt^ method^33^. The reaction product was quantified with *GAPDH* as the reference gene. For endpoint PCR, the reaction was performed with Taq polymerase (Roche Applied Science, IN, USA) in a thermocycler machine. The amplified products were electrophoresed in 1.8% agarose gel and stained with ethidium bromide. The endpoint PCR reaction started with denaturing at 95 °C for 2 min, followed by 35 cycles of amplification, and ended with an extension reaction at 72 °C for 7 min. The amplification cycles were (1) 94 °C for 45 s, (2) 60 °C for 60 s, and (3) 72 °C for 90 s. Band density was evaluated using ImageJ software. The oligonucleotide sequences used are shown in Suppl Table 1.

### MTT assay

hPDLs were incubated with 3-(4,5-dimethythiazol-2-yl)-2,5-diphenyltetrazolium bromide solution (cat. no. M6494, Invitrogen) at a final concentration of 0.5 mg/ml. The cells were maintained at 37 °C for 30 min to allow formazan crystal formation. Subsequently, the precipitated formazan was solubilized in dimethylsulfoxide/glycine buffer. The solution was measured an absorbance at 570 nm.

### ALP activity and staining

Cells were lysed with alkaline lysis buffered solution. An aliquot of the solution was subjected to ALP activity assay and total protein assay. For the ALP activity assay, an aliquot of the cell lysate was incubated with p-nitrophenol phosphate solution (PNPP, cat. no. 002201, Life technologies, Frederick, MD, USA) at 37 °C for 15 min. The reaction was stopped by adding 0.1 M NaOH. The products were measured at an absorbance of 410 nm. For total protein, an aliquot of the cell lysate was incubated with bicinchoninic acid solution (Pierce BCA protein assay kit, ThermoFisher Scientific, USA) and the assay was performed according to the manufacturer’s protocol. For ALP staining, the cells were fixed with 10% buffered formalin for 10 min. After rinsing with phosphate buffered saline, the cells were stained with BCIP/NBT tablets (Roche, USA) for 30 min and kept out from light at room temperature.

### Scanning electron microscopic and energy-dispersive X-ray spectroscopic analysis

Cells were fixed with 2.5% glutaraldehyde solution (cat. no. 49629, Sigma-Aldrich) for 10 min and dehydrated with a graded series of ethanol solutions. The samples were processed for critical point drying. The cell and mineral morphology were observed using a scanning electron microscope (SEM; Quanta 250, FEI, Netherlands). The chemical composition was determined using energy-dispersive X-ray spectroscopic analysis (EDX).

### Alizarin red s staining

Samples were fixed with cold methanol for 10 min, washed with deionized water, and stained with a 2% alizarin red s solution for 3 min at room temperature. The stained dye was solubilized with 10% cetylpyridinium chloride monohydrate in 10 mM sodium phosphate at room temperature with gently agitation for 15 min. The solubilized dye was measured an absorbance of 570 nm.

### Flow cytometry analysis

Cells were harvested to create single cell suspension and further incubated with fluorochrome labelled antibodies (dilution 1:5) for 30 min at 4 °C in PBS containing 1% FBS. The stained cells were analysed on FACSCelesta instrument (BD Bioscience). Data were presented as mean fluorescence intensity per cell. The antibodies were BB515-conjugated mouse anti-human NOTCH1 (clone MHN1-519; BD Horizon), PE-conjugated mouse anti-human NOTCH2 (clone MHN2-25; BD Pharmingen), PE-conjugated mouse anti-human NOTCH3 (clone MHN3-21; BD Pharmingen), and PE-conjugated mouse anti-human NOTCH4 (clone MHN4-2; BD Pharmingen). Fluorescence-conjugated mouse IgG was employed as the isotype control.

### Immunofluorescense staining

Cells were fixed with 4% buffered formalin for 10 min. Horse serum (2% v/v) was used to inhibit a non-specific binding. Cells were stained with mouse anti-collagen I (C2456, Sigma) at 4 °C overnight and subsequently incubated with biotinylated anti-mouse antibodies (Invitrogen) for 40 min. The targeted protein expression was visualized by stained with Strep-FITC (Sigma). DAPI (TOCRIS bioscience) was employed for nuclei staining.

### Statistical analyses

The data were presented as mean ± standard error of the mean. Each dot represents each data point. Statistical differences were assessed using the Mann Whitney U test for two group comparison or the Kruskal Wallis test followed by pairwise comparison with an adjusted *p* value for more than two group comparisons. Differences at *p* < 0.05 was considered to be significant. The statistical analysis was performed using Prism 8 (GraphPad Software, CA, USA).

## Supplementary information

Supplementary information

## References

[1] Canalis E (2013). Notch signaling in osteocytes differentially regulates cancellous and cortical bone remodeling. J. Biol. Chem..

[2] Regan J, Long F (2013). Notch signaling and bone remodeling. Curr. Osteoporos. Rep..

[3] Kikuta J, Yamaguchi M, Shimizu M, Yoshino T, Kasai K (2015). Notch signaling induces root resorption via RANKL and IL-6 from hPDL cells. J. Dent. Res..

[4] Manokawinchoke J, Sumrejkanchanakij P, Subbalekha K, Pavasant P, Osathanon T (2016). Jagged1 inhibits osteoprotegerin expression by human periodontal ligament cells. J. Periodontal. Res..

[5] Nakao A (2009). PTHrP induces Notch signaling in periodontal ligament cells. J. Dent. Res..

[6] Tanabe H (2010). Periostin associates with Notch1 precursor to maintain Notch1 expression under a stress condition in mouse cells. PLoS ONE.

[7] Osathanon T, Nowwarote N, Manokawinchoke J, Pavasant P (2013). bFGF and JAGGED1 regulate alkaline phosphatase expression and mineralization in dental tissue-derived mesenchymal stem cells. J. Cell Biochem..

[8] Osathanon T (2013). Surface-bound orientated Jagged-1 enhances osteogenic differentiation of human periodontal ligament-derived mesenchymal stem cells. J. Biomed. Mater. Res. A.

[9] Liu L, Ling J, Wei X, Wu L, Xiao Y (2009). Stem cell regulatory gene expression in human adult dental pulp and periodontal ligament cells undergoing odontogenic/osteogenic differentiation. J. Endod..

[10] Li Y, Li SQ, Gao YM, Li J, Zhang B (2014). Crucial role of Notch signaling in osteogenic differentiation of periodontal ligament stem cells in osteoporotic rats. Cell Biol. Int..

[11] Hozumi K (2020). Distinctive properties of the interactions between Notch and Notch ligands. Dev. Growth Differ..

[12] Mitsiadis TA, Lardelli M, Lendahl U, Thesleff I (1995). Expression of Notch 1, 2 and 3 is regulated by epithelial-mesenchymal interactions and retinoic acid in the developing mouse tooth and associated with determination of ameloblast cell fate. J. Cell Biol..

[13] Meester JAN (2019). Overlapping but distinct roles for NOTCH receptors in human cardiovascular disease. Clin. Genet..

[14] Nowwarote N (2018). Characterization of a bioactive Jagged1-coated polycaprolactone-based membrane for guided tissue regeneration. Arch. Oral Biol..

[15] Zhu F, Sweetwyne MT, Hankenson KD (2013). PKCdelta is required for Jagged-1 induction of human mesenchymal stem cell osteogenic differentiation. Stem Cells.

[16] Osathanon T (2013). Notch signaling is involved in neurogenic commitment of human periodontal ligament-derived mesenchymal stem cells. Stem Cells Dev..

[17] Hansamuit K, Osathanon T, Suwanwela J (2020). Effect of Jagged1 on the expression of genes in regulation of osteoblast differentiation and bone mineralization ontology in human dental pulp and periodontal ligament cells. J. Oral Biol. Craniofac. Res..

[18] Osathanon T (2019). Jagged1 promotes mineralization in human bone-derived cells. Arch. Oral Biol..

[19] Manokawinchoke J (2017). Indirect immobilized Jagged1 suppresses cell cycle progression and induces odonto/osteogenic differentiation in human dental pulp cells. Sci. Rep..

[20] Dishowitz MI (2014). Jagged1 immobilization to an osteoconductive polymer activates the Notch signaling pathway and induces osteogenesis. J. Biomed. Mater. Res. A.

[21] Osathanon T, Manokawinchoke J, Egusa H, Pavasant P (2017). Notch signaling partly regulates the osteogenic differentiation of retinoic acid-treated murine induced pluripotent stem cells. J. Oral. Sci..

[22] Shimizu T (2009). Notch signaling induces osteogenic differentiation and mineralization of vascular smooth muscle cells: role of Msx2 gene induction via Notch-RBP-Jk signaling. Arterioscler. Thromb. Vasc. Biol..

[23] Anant S, Roy S, VijayRaghavan K (1998). Twist and Notch negatively regulate adult muscle differentiation in Drosophila. Development.

[24] Zhang XW (2014). Twist-related protein 1 negatively regulated osteoblastic transdifferentiation of human aortic valve interstitial cells by directly inhibiting runt-related transcription factor 2. J. Thorac. Cardiovasc. Surg..

[25] Komaki M (2007). Twist negatively regulates osteoblastic differentiation in human periodontal ligament cells. J. Cell. Biochem..

[26] Manokawinchoke J, Sumrejkanchanakij P, Pavasant P, Osathanon T (2017). Notch signaling participates in TGF-beta-induced SOST expression under intermittent compressive stress. J. Cell Physiol..

[27] Baeten JT, Lilly B (2015). Differential regulation of NOTCH2 and NOTCH3 contribute to their unique functions in vascular smooth muscle cells. J. Biol. Chem..

[28] Baumgart A (2015). Opposing role of Notch1 and Notch2 in a Kras(G12D)-driven murine non-small cell lung cancer model. Oncogene.

[29] Yorgan T (2016). Osteoblast-specific Notch2 inactivation causes increased trabecular bone mass at specific sites of the appendicular skeleton. Bone.

[30] Ongaro A (2016). Characterization of notch signaling during osteogenic differentiation in human osteosarcoma cell line MG63. J. Cell Physiol..

[31] Chakravorty N (2014). Pro-osteogenic topographical cues promote early activation of osteoprogenitor differentiation via enhanced TGFbeta, Wnt, and Notch signaling. Clin. Oral Implants Res..

[32] Hoac B, Nelea V, Jiang W, Kaartinen MT, McKee MD (2017). Mineralization-inhibiting effects of transglutaminase-crosslinked polymeric osteopontin. Bone.

[33] Livak KJ, Schmittgen TD (2001). Analysis of relative gene expression data using real-time quantitative PCR and the 2(-Delta Delta C(T)) Method. Methods.

