# Supplementary material for: NOTCH2 participates in Jagged1-induced osteogenic differentiation in human periodontal ligament cells
Source: Sci Rep. 2020 Aug 7;10:13329. doi: 10.1038/s41598-020-70277-7 (PMC7414879; doi:10.1038/s41598-020-70277-7)
Supplement: Supplementary file 1 — Supplementary information [file 41598_2020_70277_MOESM1_ESM.docx]

**NOTCH2 participates in Jagged1-induced osteogenic differentiation in human periodontal ligament cells**

Jeeranan Manokawinchoke^1,2^, Piyamas Sumrejkanchanakij^2^, Lawan Boonprakong^3^, Prasit Pavasant^2^, Hiroshi Egusa^4^, Thanaphum Osathanon^1,2,3,*^

^1^Dental Stem Cell Biology Research Unit, Faculty of Dentistry, Chulalongkorn University, Bangkok, 10330 Thailand

^2^Center of Excellence for Regenerative Dentistry and Department of Anatomy, Faculty of Dentistry, Chulalongkorn University, Bangkok 10330 Thailand

^3^Oral Biology Research Center, Faculty of Dentistry, Chulalongkorn University, Bangkok, 10330 Thailand

^4^Division of Molecular and Regenerative Prosthodontics, Tohoku University Graduate School of Dentistry, Sendai 980-8575, JAPAN

**Running title** NOTCH2 in Jagged1-induced osteogenic differentiation

**Corresponding author**

Thanaphum Osathanon, DDS, PhD

Dental Stem Cell Biology Research Unit and Center of Excellence for Regenerative Dentistry,

Faculty of Dentistry, Chulalongkorn University, Bangkok, 10330 Thailand

Tel: +66-2-218-8885

Fax: +66-2-218-8870

Email: [thanaphum.o@chula.ac.th](mailto:thanaphum.o@chula.ac.th)

**Supplementary Table 1** The oligonucleotide sequences

| Genes | Forward sequences | Reverse sequences | Size (bp) | GenBank Accession Number | References |
| --- | --- | --- | --- | --- | --- |
| *GAPDH*  qPCR | 5’-TCATGGGTGTGAACCATGAGAA-3’ | 5’-GGCATGGACTGTGGTCATGAG-3’ | 146 | NM_002046.3 | (1) |
| *GAPDH*  Conventional PCR | 5’-TGAAGGTCGGAGTCAACGGAT-3’ | 5’-TCACACCCATGACGAACATGG-3’ | 396 | NM_002046.3 | (2) |
| *MSX2* | 5’-GGTTTCCTCTCCCTCTCCAC-3’ | 5’-GGCTTGGTGCCTCCGCCTAC-3’ | 140 | NM_002449.4 |  |
| *COL1A1* | 5’-GTGCTAAAGGTGCCAATGGT-3’ | 5’-ACCAGGTTCACCGCTGTTAC-3’ | 128 | NM_000088.4 | (3) |
| *ALP* | 5’-CGAGATACAAGCACTCCCACTTC-3’ | 5’-CTGTTCAGCTCGTACTGCATGTC-3’ | 120 | NM_000478.3 | (4) |
| *RUNX2* | 5’-ATGATGACACTGCCACCTCTGA-3’ | 5’-GGCTGGATAGTGCATTCGTG-3’ | 167 | NM_001024630.3 | (5) |
| *OSX* | 5’-GCCAGAAGCTGTGAAACCTC-3’ | 5’-GCTGCAAGCTCTCCATAACC-3’ | 161 | NM_001173467.3 | (6) |
| *OCN* | 5’-CTTTGTGTCCAAGCAGGAGG-3’ | 5’-CTGAAAGCCGATGTGGTCAG-3’ | 166 | NM_199173.4 | (7) |
| *TWIST1* | 5’-TCTTACGFGGAGCTGCAGACGCA-3’ | 5’-ATCTTGGAGTCCAGCTCGTCG-3’ | 212 | NM_000474.3 | (8) |
| *TWIST2* | 5’-GCTGCGCAAGATCATCCC-3’ | 5’-GTAGCTGCAGCTGGTCATC-3’ | 142 | NM_057179.3 | (8) |
| *ENPP* | 5’-CACAAGAAACCCCAGAGATAAC-3’ | 5’-CCACTGACGACATTGACAC-3’ | 540 | NM_006208.3 | (9) |
| *ANKH* | 5’-GAGGTGACAGACATCGTGG-3’ | 5’-CCTTTAAATCAAGGCCTCTTTCA-3’ | 177 | NM_054027.6 | (9) |
| *PIT1* | 5’-GGAGGGTGTCAAGTGGTCTGAA-3’ | 5’-ATCTGCCTTATGGAGGATGAATG-3’ | 127 | NM_005415.5 | (10) |
| *OPN* | 5’-AGGAGGAGGCAGAGCACA-3’ | 5’-CTGGTATGGCACAGGTGATG-3’ | 150 | NM_001040058.2 | (11) |
| *DMP1* | 5’- CAGGAGCACAGGAAAAGGAG -3’ | 5’- CTGGTGGTATCTTGGGCACT -3’ | 213 | NM_004407.3 | (12) |
| *DSPP* | 5’-ATATTGAGGGCTGGAATGGGGA-3’ | 5’-TTTGTGGCTCCAGCATTGTCA-3’ | 136 | NM_014208.3 | (13) |
| *NOTCH1* | 5’-AGGACCTCATCAACTCACACGC-3’ | 5’-TCTTTGTTAGCCCCGTTCTTCAG-3’ | 130 | NM_017617.5 | (14) |
| *NOTCH2* | 5’-CCGTGTTGACTTCTGCTCTCTA-3’ | 5’-CCTACTACCCTTGGCATCCTTTG-3’ | 170 | NM_024408.4 | (14) |
| *NOTCH3* | 5’-TCTCAGACTGGTCCGAATCCAC-3’ | 5’-ACACTTGCCTCTTGGGGGTAAC-3’ | 171 | NM_000435.3 | (14) |
| *NOTCH4* | 5’-ATGCGAGGAAGATACGGAGTGG-3’ | 5’-TCGGAATGTTGGAGGCAGAAC-3’ | 112 | NM_004557.4 | (14) |
| *HES1* | 5’-AGGCGGACATTCTGGAAATG-3’ | 5’-CGGTACTTCCCCAGCACACTT-3’ | 103 | NM_005524.4 | (15) |
| *HEY1* | 5’-CTGCAGATGACCGTGGATCA-3’ | 5’-CCAAACTCCGATAGTCCATAGCA-3’ | 97 | NM_012258.4 | (16) |
| *JAG1* | 5’-ACTGCTCACACCTGAAAGACCAC-3’ | 5’-AGGACCACAGACGTTGGAGGAAA-3’ | 128 | NM_000214.3 | (17) |
| *JAG2* | 5’-TCGGGCAGGAACTGTGAGAAGGC-3’ | 5’-AATCACAGTAATAGCCGCCAATCAGGT-3’ | 328 | NM_002226.5 | (18) |
| *DLL1* | 5’-TGTGACGAGTGTATCCGCTATCC-3’ | 5’-AGGGCTTATGGTGTGTGCAGTAG-3’ | 133 | NM_005618.4 | (17) |
| *DLL3* | 5’-TCCCGGATGCACTCAACAACCTA-3’ | 5’-TTCAGGGCGATTCCAATCTACGG-3’ | 83 | NM_016941.4 | (17) |
| *DLL4* | 5’-ACTGCGAGAAGAAAGTGGACAGG-3’ | 5’-ACATGAGCCCATTCTCCAGGTCA-3’ | 198 | NM_019074.4 | (17) |

**References**

1. Li R, Li X, Zhou M, Han N, Zhang Q. Quantitative determination of matrix Gla protein (MGP) and BMP-2 during the osteogenic differentiation of human periodontal ligament cells. Arch Oral Biol. 2012;57(10):1408-17.

2. Wongkhantee S, Yongchaitrakul T, Pavasant P. Mechanical stress induces osteopontin via ATP/P2Y1 in periodontal cells. J Dent Res. 2008;87(6):564-8.

3. Ono Y, Hayashida T, Konagai A, Okazaki H, Miyao K, Kawachi S, et al. Direct inhibition of the transforming growth factor-beta pathway by protein-bound polysaccharide through inactivation of Smad2 signaling. Cancer Sci. 2012;103(2):317-24.

4. Xu WP, Mizuno N, Shiba H, Takeda K, Hasegawa N, Yoshimatsu S, et al. Promotion of functioning of human periodontal ligament cells and human endothelial cells by nerve growth factor. J Periodontol. 2006;77(5):800-7.

5. Limjeerajarus CN, Chanarattanubol T, Trongkij P, Rujiwanichkul M, Pavasant P. Iloprost induces tertiary dentin formation. J Endod. 2014;40(11):1784-90.

6. Valenzuela CD, Allori AC, Reformat DD, Sailon AM, Allen RJ, Jr., Davidson EH, et al. Characterization of adipose-derived mesenchymal stem cell combinations for vascularized bone engineering. Tissue Eng Part A. 2013;19(11-12):1373-85.

7. Galea GL, Sunters A, Meakin LB, Zaman G, Sugiyama T, Lanyon LE, et al. Sost down-regulation by mechanical strain in human osteoblastic cells involves PGE2 signaling via EP4. FEBS Lett. 2011;585(15):2450-4.

8. Isenmann S, Arthur A, Zannettino AC, Turner JL, Shi S, Glackin CA, et al. TWIST family of basic helix-loop-helix transcription factors mediate human mesenchymal stem cell growth and commitment. Stem cells. 2009;27(10):2457-68.

9. Rodrigues TL, Foster BL, Silverio KG, Martins L, Casati MZ, Sallum EA, et al. Hypophosphatasia-associated deficiencies in mineralization and gene expression in cultured dental pulp cells obtained from human teeth. J Endod. 2012;38(7):907-12.

10. Li X, Yang HY, Giachelli CM. Role of the sodium-dependent phosphate cotransporter, Pit-1, in vascular smooth muscle cell calcification. Circ Res. 2006;98(7):905-12.

11. Chan RW, Ng EH, Yeung WS. Identification of cells with colony-forming activity, self-renewal capacity, and multipotency in ovarian endometriosis. Am J Pathol. 2011;178(6):2832-44.

12. Cavalcanti BN, Zeitlin BD, Nor JE. A hydrogel scaffold that maintains viability and supports differentiation of dental pulp stem cells. Dent Mater. 2013;29(1):97-102.

13. Gong W, Huang Z, Dong Y, Gan Y, Li S, Gao X, et al. Ionic extraction of a novel nano-sized bioactive glass enhances differentiation and mineralization of human dental pulp cells. J Endod. 2014;40(1):83-8.

14. Watanabe K, Nagaoka T, Lee JM, Bianco C, Gonzales M, Castro NP, et al. Enhancement of Notch receptor maturation and signaling sensitivity by Cripto-1. J Cell Biol. 2009;187(3):343-53.

15. Muller P, Kietz S, Gustafsson JA, Strom A. The anti-estrogenic effect of all-trans-retinoic acid on the breast cancer cell line MCF-7 is dependent on HES-1 expression. The Journal of biological chemistry. 2002;277(32):28376-9.

16. Wang H, Chen Y, Fernandez-Del Castillo C, Yilmaz O, Deshpande V. Heterogeneity in signaling pathways of gastroenteropancreatic neuroendocrine tumors: a critical look at notch signaling pathway. Mod Pathol. 2013;26(1):139-47.

17. Monsalve EM, Garcia-Gutierrez MS, Navarrete F, Giner S, Laborda J, Manzanares J. Abnormal expression pattern of Notch receptors, ligands, and downstream effectors in the dorsolateral prefrontal cortex and amygdala of suicidal victims. Mol Neurobiol. 2014;49(2):957-65.

18. Xiao W, Gao Z, Duan Y, Yuan W, Ke Y. Notch signaling plays a crucial role in cancer stem-like cells maintaining stemness and mediating chemotaxis in renal cell carcinoma. J Exp Clin Cancer Res. 2017;36(1):41.
